# Supplementary material for: The test-retest reliability and agreement between a fixed frame and belt-stabilised handheld dynamometer for isometric hip flexion and extension peak force measurement in recreational cyclists
Source: PLoS One. 2026 Apr 3;21(4):e0328143. doi: 10.1371/journal.pone.0328143 (PMC13048430; doi:10.1371/journal.pone.0328143)
Supplement: S2 Appendix — (DOCX) [file pone.0328143.s002.docx]

**The test-retest reliability and agreement between a fixed frame and belt-stabilised handheld dynamometer for isometric hip flexion and extension peak force measurement in recreational cyclists**

(**S2 Appendix** – Testing positions)

Dion D’Mello ^1^

Benn Digweed ^1, 2^

Tom Hughes ^1, 3^

**Affiliations:**

^1^Department of Health Professions, Manchester Metropolitan University, Manchester, UK.

^2^ United Kingdom Sports Institute, UK Sports Institute High Performance Centre, Manchester Institute of Health and Performance, Manchester, UK.

^3^Institute of Sport, Manchester Metropolitan University, Manchester, UK.

**Corresponding author:**

Tom Hughes

Email: t.hughes@mmu.ac.uk

ORCID ID: 0000-0003-2266-6615


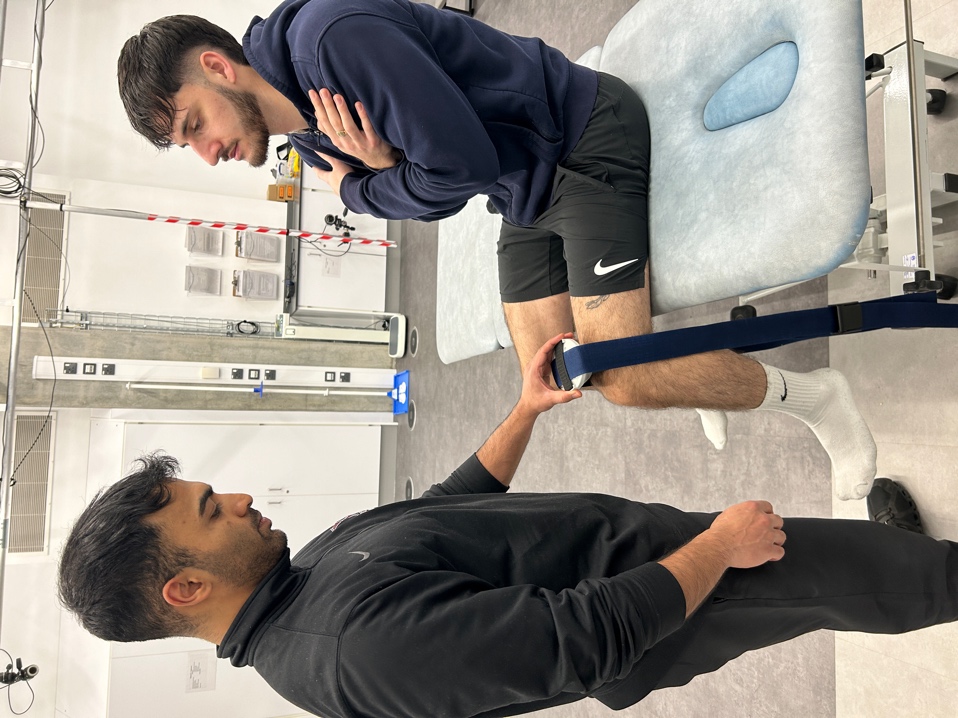


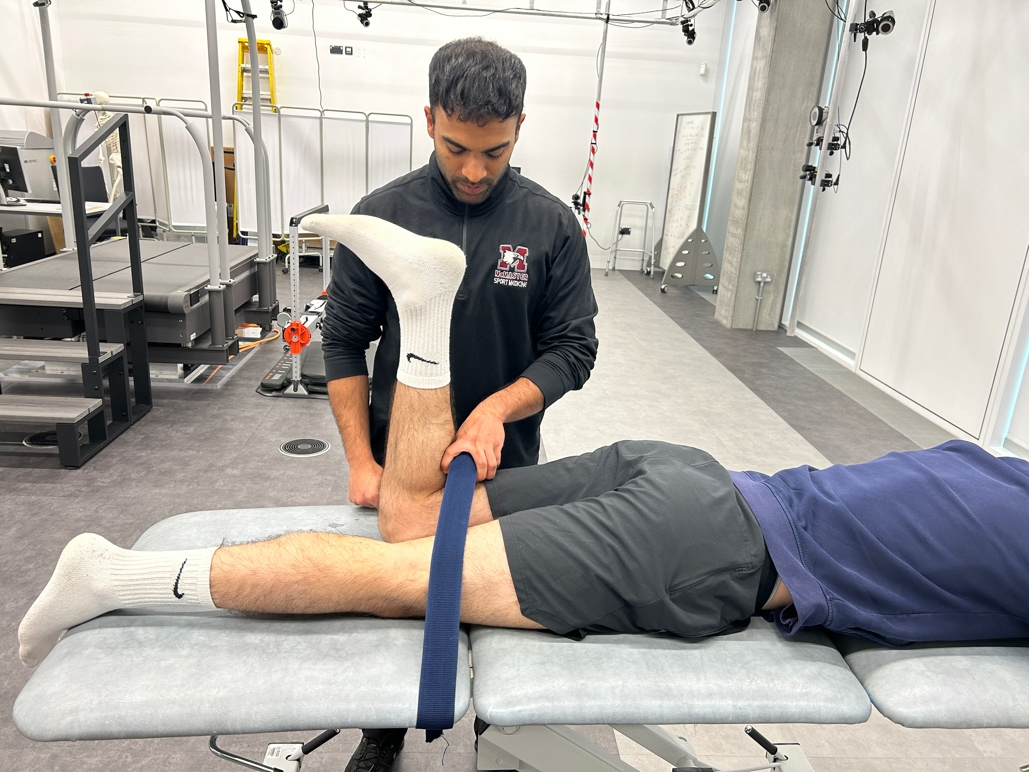


B) Participants laid prone on an examination table with their hands under their forehead, knee of the leg being tested flexed to 90 degrees, and the transducer placed 1 inch superior to the popliteal fossa. The examiner was only present to ensure the handheld dynamometer did not slip.

A) Participants were seated upright on an examination table with their arms crossed against their chest, hips flexed to 90 degrees and the transducer placed 1 inch superior to the base of the patella. The examiner was only present to ensure the handheld dynamometer did not slip.


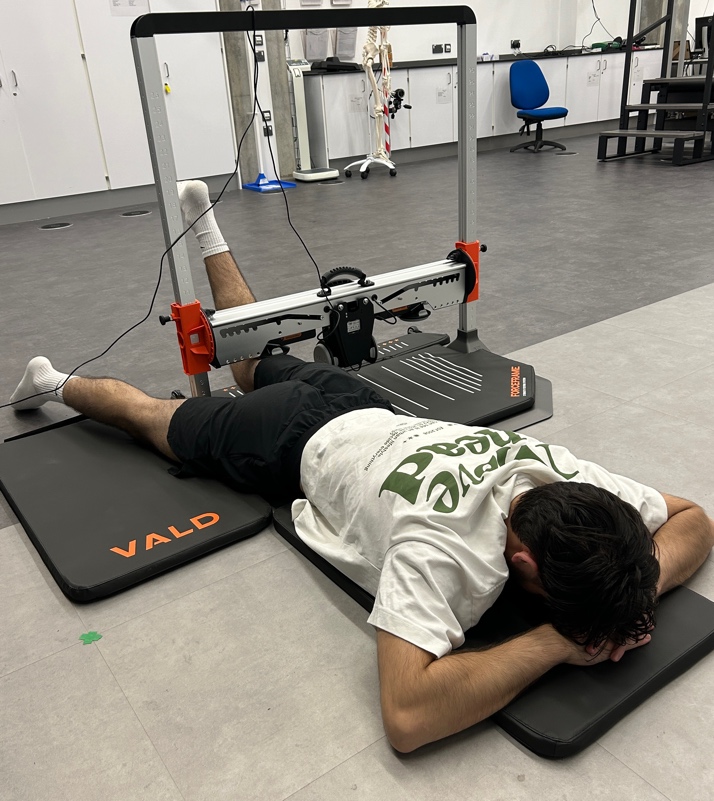


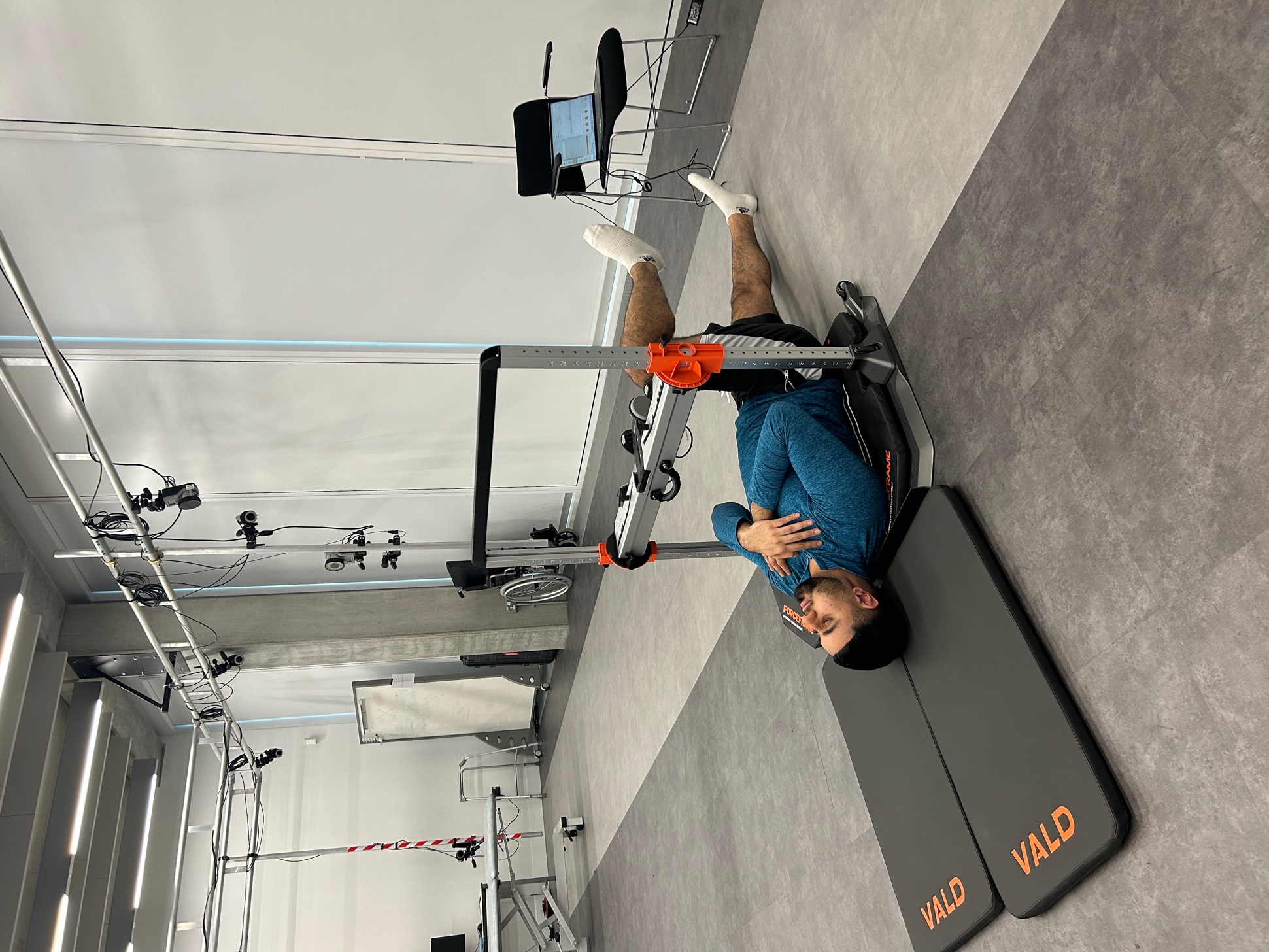


C) Participants laid supine on the floor with their arms crossed against their chest, hip of the leg being tested flexed to 90 degrees, and height of the ForceFrame adjusted to allow the load cell to be positioned 1 inch superior to the base of the patella.

D) Participants laid prone on the floor with their hands under their forehead, knee of the leg being tested flexed to 90 degrees, and height of the ForceFrame adjusted to allow the load cell to be positioned 1 inch superior to the popliteal fossa.
