# Supplementary material for: The test-retest reliability and agreement between a fixed frame and belt-stabilised handheld dynamometer for isometric hip flexion and extension peak force measurement in recreational cyclists
Source: PLoS One. 2026 Apr 3;21(4):e0328143. doi: 10.1371/journal.pone.0328143 (PMC13048430; doi:10.1371/journal.pone.0328143)
Supplement: S3 Appendix — (DOCX) [file pone.0328143.s003.docx]

#

# **The test-retest reliability and agreement between a fixed frame and belt-stabilised handheld dynamometer for isometric hip flexion and extension peak force measurement in recreational cyclists**

(**S3 Appendix** – **Quantile-Quantile Plots to show distribution of raw scores; Histograms to show the distribution of residuals, for both devices, across limb, testing day and muscle groups.**)

Dion D’Mello ^1^

Benn Digweed ^1, 2^

Tom Hughes ^1, 3^

**Affiliations:**

^1^Department of Health Professions, Manchester Metropolitan University, Manchester, UK.

^2^ United Kingdom Sports Institute, UK Sports Institute High Performance Centre, Manchester Institute of Health and Performance, Manchester, UK.

^3^Institute of Sport, Manchester Metropolitan University, Manchester, UK.

**Corresponding author:**

Tom Hughes

Email: t.hughes@mmu.ac.uk

ORCID ID: 0000-0003-2266-6615

**1.a) Distribution of raw scores for hip flexion using the ForceFrame**


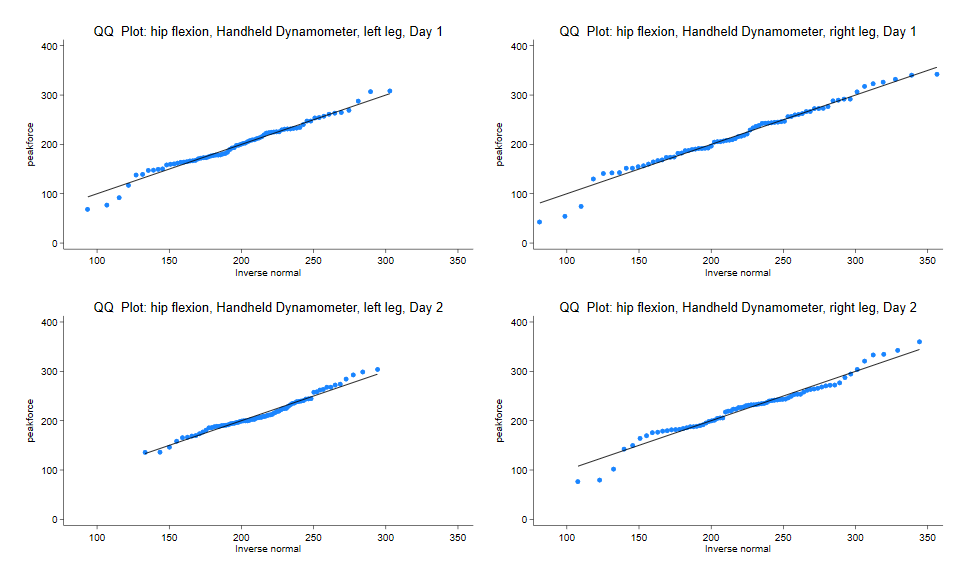


**1.b) Distribution of raw scores for hip flexion using the Handheld Dynamometer**
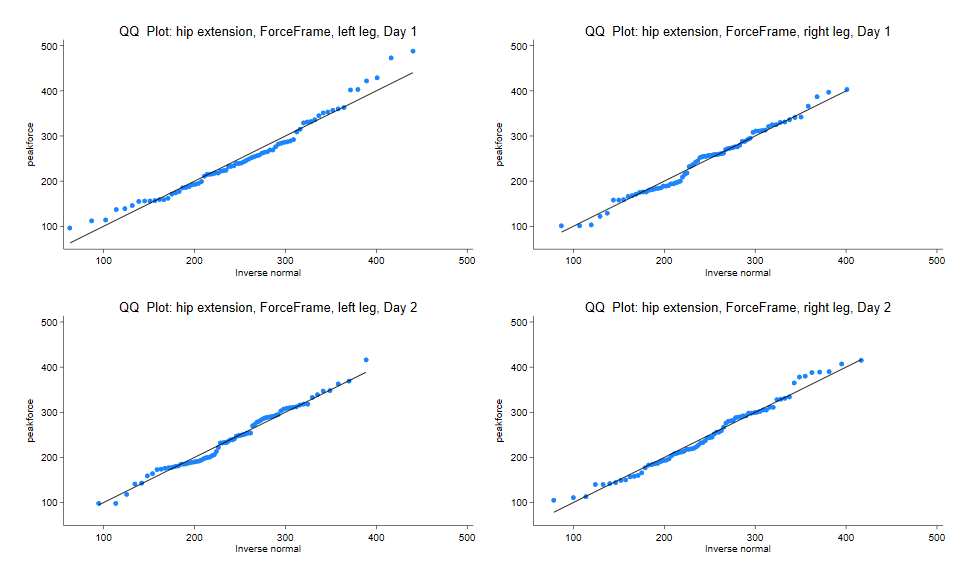


**1.c) Distribution of raw scores for hip extension using the ForceFrame**
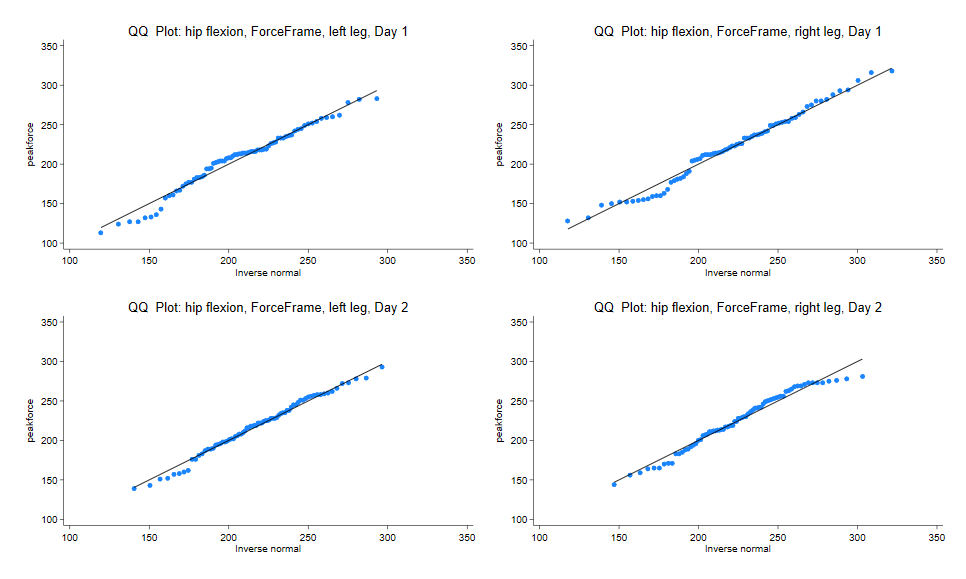


**1.d) Distribution of raw scores for hip extension using the Handheld Dynamometer
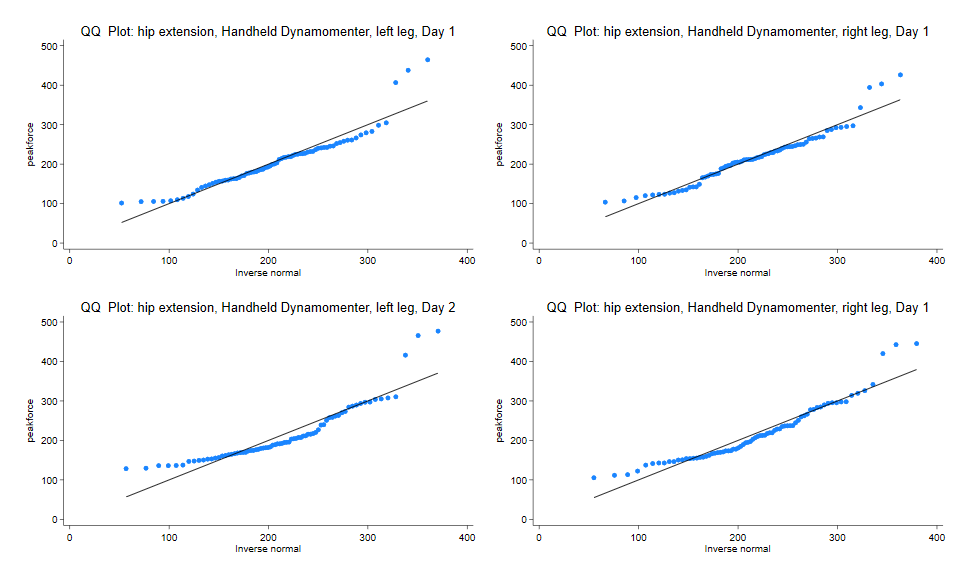
**

**2.a) Distribution of residuals for hip flexion using the ForceFrame**


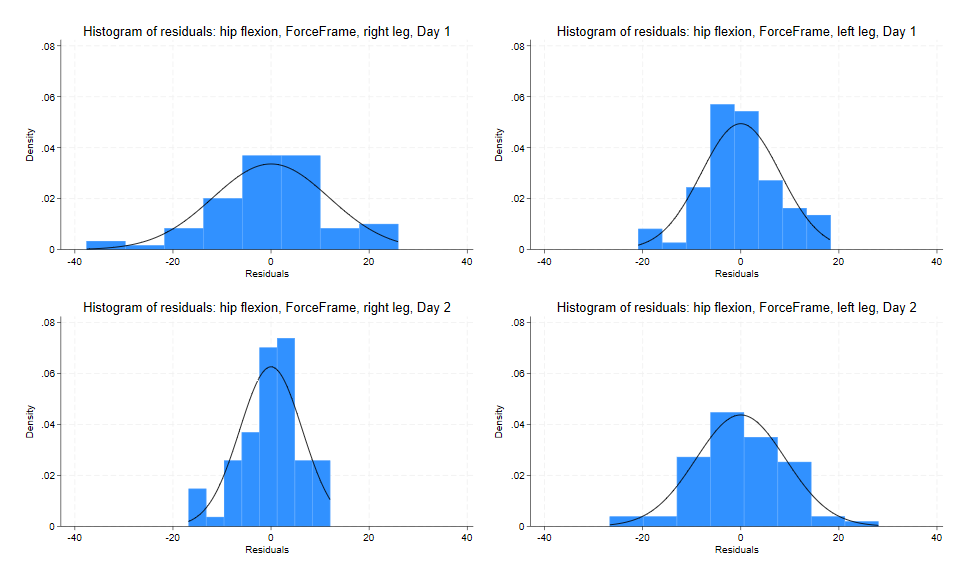


**2.b) Distribution of residuals for hip flexion using the Handheld Dynamometer
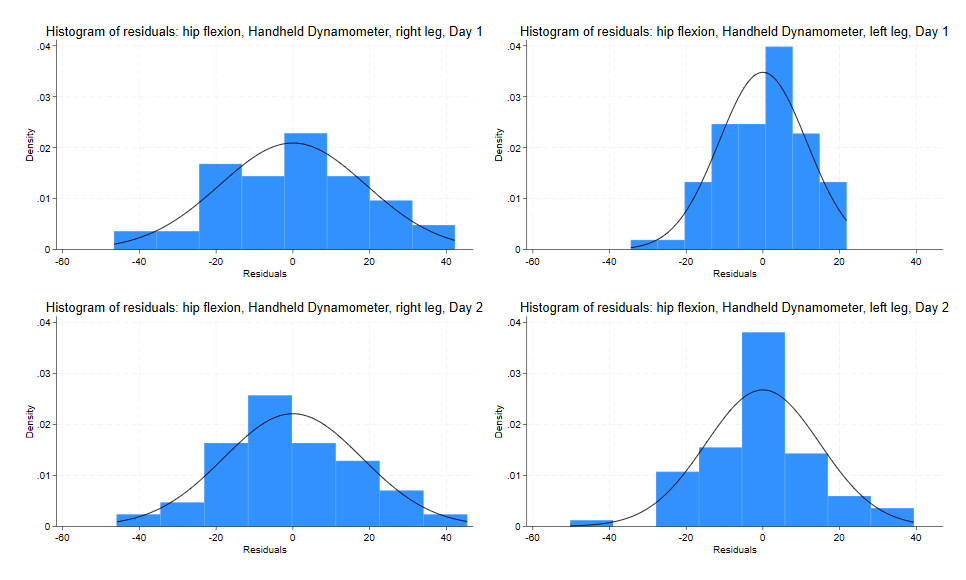
**

**2.c) Distribution of residuals for hip extension using the ForceFrame**
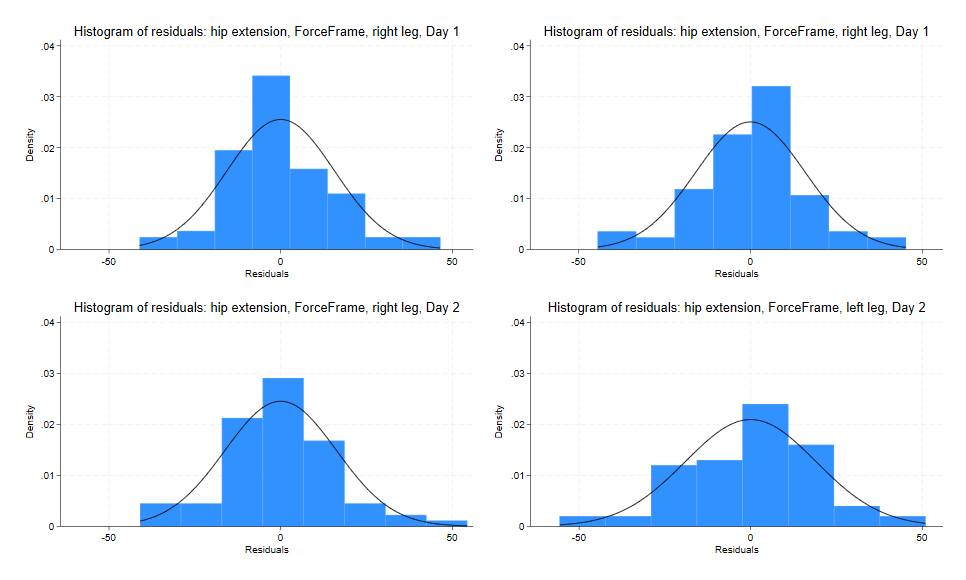


**2.d) Distribution of residuals for hip extension using the Handheld Dynamometer** **
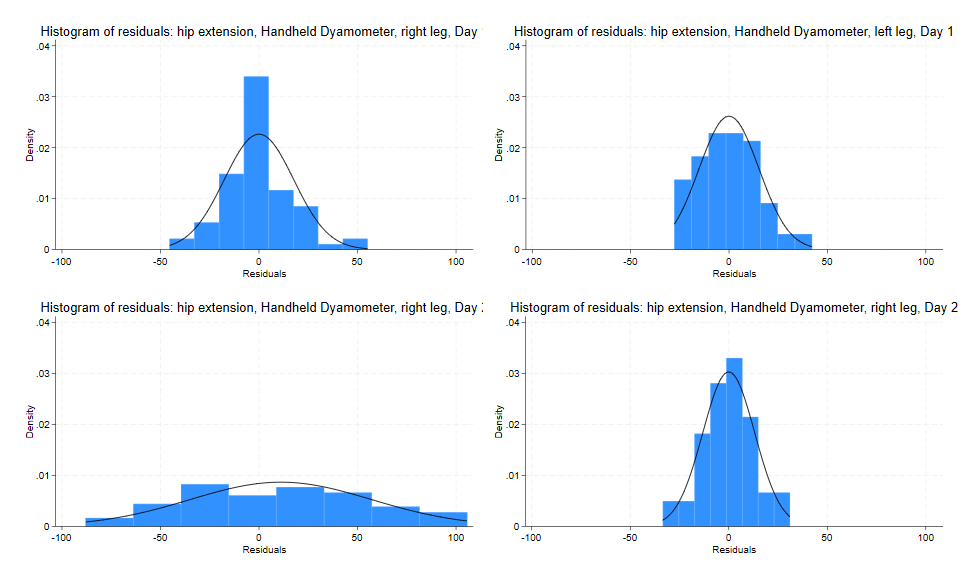
**
