# Supplementary material for: The test-retest reliability and agreement between a fixed frame and belt-stabilised handheld dynamometer for isometric hip flexion and extension peak force measurement in recreational cyclists
Source: PLoS One. 2026 Apr 3;21(4):e0328143. doi: 10.1371/journal.pone.0328143 (PMC13048430; doi:10.1371/journal.pone.0328143)

# **The test-retest reliability and agreement between a fixed frame and belt-stabilised handheld dynamometer for isometric hip flexion and extension peak force measurement in recreational cyclists**

(**S4 Appendix** – Quantile-quantile plots to show distribution of between device differences across limb, testing day and muscle groups)

Dion D’Mello ^1^

Benn Digweed ^1, 2^

Tom Hughes ^1, 3^

**Affiliations:**

^1^Department of Health Professions, Manchester Metropolitan University, Manchester, UK.

^2^ United Kingdom Sports Institute, UK Sports Institute High Performance Centre, Manchester Institute of Health and Performance, Manchester, UK.

^3^Institute of Sport, Manchester Metropolitan University, Manchester, UK.

**Corresponding author:**

Tom Hughes

Email: t.hughes@mmu.ac.uk

ORCID ID: 0000-0003-2266-6615


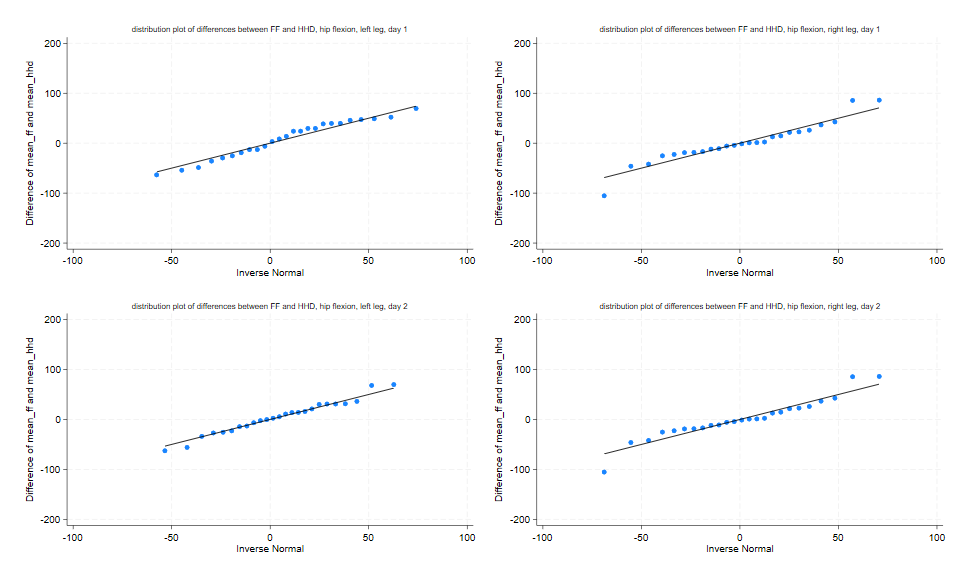

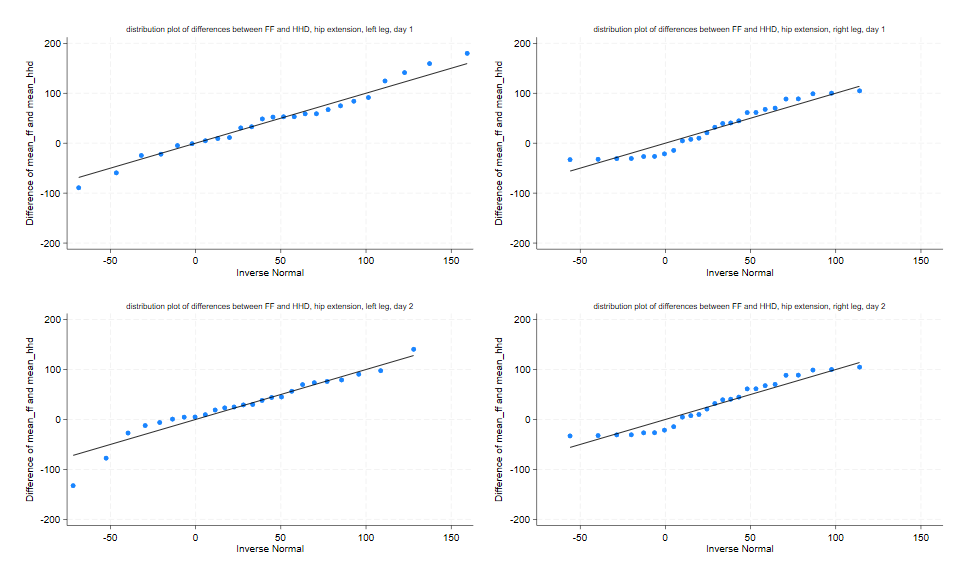

Supplement: S4 Appendix — (DOCX) [file pone.0328143.s004.docx]
