# Supplementary material for: The test-retest reliability and agreement between a fixed frame and belt-stabilised handheld dynamometer for isometric hip flexion and extension peak force measurement in recreational cyclists
Source: PLoS One. 2026 Apr 3;21(4):e0328143. doi: 10.1371/journal.pone.0328143 (PMC13048430; doi:10.1371/journal.pone.0328143)
Supplement: Attachment — Submitted filename: Response to reviewers.docx [file pone.0328143.s007.docx]

Tom Hughes
Department of Health Professions
Manchester Metropolitan University
Email: t.hughes@mmu.ac.uk

Dear Dr Ahmed

Re: Revisions required for manuscript entitled: The test-retest reliability and agreement between a fixed frame and belt-stabilised handheld dynamometer for isometric hip flexion and extension peak force measurement in recreational cyclists (PONE-D-25-34937)

We would like to take the opportunity to extend our thanks to you and the reviewers for taking the time to consider our manuscript for publication in PLOS ONE. We are encouraged by the favourable response and have taken time to consider the feedback provided. We have now addressed these issues and feel that the manuscript offers a stronger submission as a result.

Please find our replies (in **bold** type) to the reviewer’s comments (in *italic* type), which explain how they have been dealt with in the paper. All amended text in the manuscript has been highlighted in yellow. We have copied and pasted excerpts of the text in the replies below where appropriate, to identify the changes made. Please note that line numbers referenced in the response relate to the Revised Manuscript with Tracked Changes.

Editor Comments

*1.Please ensure that your manuscript meets PLOS ONE's style requirements, including those for file naming. The PLOS ONE style templates can be found at*

[*https://journals.plos.org/plosone/s/file?id=wjVg/PLOSOne_formatting_sample_main_body.pdf*](https://url.uk.m.mimecastprotect.com/s/5A7WC6m8rcrlqNNxH5sDt5vWQU?domain=journals.plos.org) *and*

[*https://journals.plos.org/plosone/s/file?id=ba62/PLOSOne_formatting_sample_title_authors_affiliations.pdf*](https://url.uk.m.mimecastprotect.com/s/040rC7n3vuAjwgg9UqtBtoGU-Q?domain=track.editorialmanager.com)

**Thank you for highlighting this. We have now amended the title page and adjusted formatting precisely throughout the document to ensure the manuscript conforms to publishing guidelines**.

*2. Thank you for stating the following in the Competing Interests section:*

*“DDM was a student at Manchester Metropolitan University and this work was submitted as a dissertation project for the qualification of MSc Physiotherapy. He was employed at Manchester Movement Unit and British Cycling as a Sports Massage therapist during data collection. The project was conceived during his employment with British Cycling. BD was employed by Manchester Metropolitan University and the UK Sports Institute. TH is employed at Manchester Metropolitan University.”*

*Please confirm that this does not alter your adherence to all PLOS ONE policies on sharing data and materials, by including the following statement: "This does not alter our adherence to  PLOS ONE policies on sharing data and materials.” (as detailed online in our guide for authors* [*http://journals.plos.org/plosone/s/competing-interests*](https://url.uk.m.mimecastprotect.com/s/sBXMC8o2wU68EGGxIwulty5iqr?domain=journals.plos.org)*).  If there are restrictions on sharing of data and/or materials, please state these. Please note that we cannot proceed with consideration of your article until this information has been declared.*

**We apologise for the omission of this statement. As requested, we have included the statement in the ‘Competing Interests’ section (line 627) which now states:**

**“DDM was a student at Manchester Metropolitan University and this work was submitted as a dissertation project for the qualification of MSc Physiotherapy. He was employed at Manchester Movement Unit and British Cycling as a Sports Massage therapist during data collection. The was conceived during his employment with British Cycling. BD was employed by Manchester Metropolitan University and the UK Sports Institute. TH is employed at Manchester Metropolitan University. This does not alter our adherence to PLOS ONE policies on sharing data and materials.”**

*Please include your full ethics statement in the ‘Methods’ section of your manuscript file. In your statement, please include the full name of the IRB or ethics committee who approved or waived your study, as well as whether or not you obtained informed written or verbal consent. If consent was waived for your study, please include this information in your statement as well.*

**Thank you for your comment and we apologise if this was unclear in the submitted document. This was already in place in the ‘Methods’ section, under the subheading of ‘Participants’. For clarity, we have also added that the study was conducted in accordance with the Declaration of Helskini in 2013, as data collection preceded publication of the updated Declaration on 19 October 2024. This text now states (line 214):**

**“Ethical approval was granted by the Faculty of Health and Education Research Ethics and Governance Committee at Manchester Metropolitan University (no.59674) and conducted in accordance with the 2013 Declaration of Helsinki. All participants provided written informed consent prior to participation.”**

If the reviewer comments include a recommendation to cite specific previously published works, please review and evaluate these publications to determine whether they are relevant and should be cited. There is no requirement to cite these works unless the editor has indicated otherwise.

**The reviewers have not recommended any citations to be added, so no action is needed here.**

*Please review your reference list to ensure that it is complete and correct. If you have cited papers that have been retracted, please include the rationale for doing so in the manuscript text or remove these references and replace them with relevant current references. Any changes to the reference list should be mentioned in the rebuttal letter that accompanies your revised manuscript. If you need to cite a retracted article, indicate the article’s retracted status in the References list and also include a citation and full reference for the retraction notice.*

**All references included have been checked again for completeness on 8^th^ October 2025. No papers included have a retracted status. In relation to addressing a comment from Reviewer 1, we have added in a new reference (see below)**

*When completing the data availability statement of the submission form, you indicated that you will make your data available on acceptance. We strongly recommend all authors decide on a data sharing plan before acceptance, as the process can be lengthy and hold up publication timelines. Please note that, though access restrictions are acceptable now, your entire data will need to be made freely accessible if your manuscript is accepted for publication. This policy applies to all data except where public deposition would breach compliance with the protocol approved by your research ethics board. If you are unable to adhere to our open data policy, please kindly revise your statement to explain your reasoning and we will seek the editor's input on an exemption. Please be assured that, once you have provided your new statement, the assessment of your exemption will not hold up the peer review process.*

**Apologies for this; our data sharing plan has already been agreed, hence the reason why we have included our raw data and code; this has now been amended on the submission form.**

**Reviewer 1 Comments**

*Study question is clear, but several issues limit the strength of the contribution, particularly in methodology, clarity of presentation, and robustness of conclusions
The Introduction does not clearly highlight the specific gap in the literature that this study addresses.
What does this study add that was not previously known?*

**Thank you for taking the time and effort to review our paper, and we appreciate your feedback.**

**In relation to your point made above, throughout the introduction, we have explicitly outlined strong reasons for why this study is needed.**

**For example, on line 135, we highlight that because hip strength influences cycling performance, strength evaluation is important:**

**“Given the importance of hip strength in cycling performance and the risk of injury associated with cycling participation (****18****), regular muscle strength assessments may provide valuable data for cyclists, coaches and health professionals. Indeed, regular physical testing can be useful for performance tracking, training evaluation and planning (****19-21****), setting rehabilitation targets (****19****) and assessing treatment effectiveness following injury (****22****).”**

**On line 159, to justify investigating the use of belt stabilised handheld dynamometers (B-HHDs) in cyclists we explicitly state that:**

**“Importantly, population characteristics influence device reliability (****31****,** **32****), but to date, the reliability of external, belt-stabilised HHDs (B-HHDs) has not been investigated in cyclists, so it is unclear if these estimates apply to this population.”**

**We also** **justify the need to investigate fixed frame ForceFrame (FF) devices in cyclists (line 164):**

**“While belt stabilisation may improve reliability, newer fixed frame dynamometers (such as the Vald ForceFrame (herein termed FF) Max (Vald Performance, Brisbane, Australia)), may offer superior stabilisation because sensors are mounted directly to a frame and base. Fixed frame devices are also highly adjustable, which allows testing of most muscle groups. A different iteration of the FF (i.e. ForceFrame Fold) has shown good to excellent test-retest reliability for hip adduction (ICC_2.1_= 0.81-0.90) and moderate to excellent for hip abduction MVICs (ICC _2.1_= 0.72-0.92) in field sport athletes (****33****). However, to the best of our knowledge, no previous studies have investigated the reliability of any FF version for measuring hip flexion and extension MVICs in any population.”**

**Finally, given the increased use of fixed frame devices to replace traditionally used B-HHDs in practice, we specifically state that (line 175):**

**“Given the growing preference to replace HHDs with fixed frame devices, (****34****) it is essential to determine whether measurements from both devices can be interchanged. This may help to ensure consistent and accurate data in settings where both devices are available, where longitudinal data have already been collected by B-HHDs, or when practitioners are deciding whether to use one method over another.**

**The aim of this study was to evaluate the test-retest reliability and agreement between a belt-stabilised MicroFET2 HHD and the ForceFrame Max, for measurement of hip flexion and extension MVIC peak force in recreational cyclists.”**

**Consequently, we feel that we have strongly justified and reasoned why this study is needed, and why its novelty will contribute to the existing evidence base. We therefore feel no change is necessary here.**

*No sample size/power calculation is provided.*

**Thank you for your comment. However, we have already specifically provided a sample size calculation (S1 Appendix), and there is a dedicated explanation of sample size in the Methods section on line 219, which states:**

**“Sample size was calculated using the confidence interval (CI) width procedure of the ICC & SEM Power Sample Size Decision Assistant (**[**https://iriseekhout.shinyapps.io/ICCpower**](https://iriseekhout.shinyapps.io/ICCpower)**) *(******38******)*. Previously reported intraclass correlation coefficents (ICCs) for hip flexion and extension strength measurements were 0.91 and 0.90 for HHDs with external stabilisation (****27****) and 0.77- 0.86 for the Groinbar, (Vald Performance, Brisbane, Australia) (****23****) a similar fixed frame device to the Forceframe Max used in this study. For a two-way mixed effects ICC model needed for a test-retest design, our calculation assumed a conservative expected correlation of 0.80, a moderate variance of 10 for between-participant test scores with no expected systematic differences, and aimed for a target CI width of 0.30. Our calculation indicated that recruitment of 25 participants would enable ICC estimation with CI precision of 0.26 for within-session analyses (three trials per testing session), and 0.33 for between-session analyses (mean of three trials are compared over two testing sessions) (see S1 Appendix for full calculation).”**

**Therefore, we feel no further action is required.**

*Which covariates were adjusted for, rationale for chosen tests, handling of missing data*

**Thank you for your comment. In accordance with the COnsensus-based Standards for the selection of health Measurement INstruments (COSMIN) Reporting Guideline (Gagnier et al, 2021), COSMIN Risk of Bias Tool (Mokkink et al, 2021) and its Guidance for Systematic Reviews of Patient-Reported Outcome Measures (Mokkink et al, 2024), adjustment for covariates is neither required nor recommended in studies assessing reliability. COSMIN advocates for the use of intraclass correlation coefficients (ICCs) to evaluate reliability for continuous data, which we deemed appropriate for our analysis. ICCs are designed to capture the proportion of total variance attributable to differences between subjects or clusters. Introducing covariate adjustment can artificially reduce this variance, resulting in context-specific estimates that may not accurately reflect the underlying reliability of the measure. For this reason, we followed COSMIN’s recommendation to report unadjusted ICCs. Adjusted ICCs may be appropriate in exploratory analyses or when assessing sources of heterogeneity, but they are not standard for primary reliability reporting.**

**In terms of the rationale for ICC model selection, we apologise that this is unclear. We have amended this and added a reference to support our choice (Koo and Li, 2016). This can be found in two locations: line 226, which now states:**

**“For a two-way mixed effects ICC model that is most suitable for a test-retest design used in this study , our calculation assumed a conservative expected correlation of 0.80, a moderate variance of 10 for between-participant test scores with no expected systematic differences, and aimed for a target CI width of 0.30.”**

**This was also reiterated this in the statistical analysis section (line 304):**

**“Two-way mixed effects ICC models are required for a test-retest design (****39****) used in this study, so within session ICCs_3.1_ and between session ICCs_3.k_ were calculated with corresponding 95% CIs and interpreted as poor < 0.40; fair= 0.40- 0.70; good=0.70-0.90; excellent > 0.90 (****40****)”.**

**In terms of missing data, only one particpant withdrew and this is clearly stated in the results, but we have specifically amended this to clarify that there is no other missing data (line 326):**

**“Of twenty-six participants recruited, one withdrew due to pain during testing and was excluded from the analysis. In the remaining dataset, there were no instances of missing data.”**

*Tables/figures could be improved for clarity: labels are sometimes unclear; some redundancy exists between text and figures*

**Thank you for highlighting this. We agree that the units of measurement could be clearer. We have now amended the captions for Table 1, which now states (line 346):**

**“Table 1: Within-session descriptive statistics for peak force measurements, per device and testing day.”**

**The caption now states (line 352):**

**“Key: B-HHD = belt stabilised handheld dynamometer; FF = Forceframe; SD = standard deviation. Please note that all measurements are in newtons (N).”**

**The title of Table 2 has also been clarified and now states (Line 361):**

**“Table 2: Within-session ICCs_3.1,_ 95%CIs, SEM and MDC estimates for both devices and limbs, according to testing day”.**

**Table 3 has also been clarified; on review of the submitted manuscript there appears to have been an issue with the text alignment. This has now been resolved and the title has now been amended, which now states (line 396):**

**“Table 3: Between-session descriptive statistics, ICCs, 95%CIs, SEM and MDC estimates for both devices, according to limb.”**

*Conclusions sometimes overstate findings relative to the data presented*

**Thank you for your feedback. We have taken care to try not to overstate the findings of the study, and this is especially important given the restricted generalisability due to the two-way mixed ICC models used.**

**Examples of where we have been careful with terminology include:**

**Line 496: “These findings tentatively suggest that different FF models may perform consistently across populations and hip muscle groups, though further studies are needed to confirm this.”**

**Line 514: “Collectively, these findings suggest that while both devices in our study are reliable for hip extension MVIC peak force measurement, the close consistency with other dynamometers, muscle groups and measurement parameters may be a function of the stable prone test position for hip extension, but this should be also be confirmed in future studies”.**

**Line 524: “Our observed MDC values for both devices suggest potential practical limitations for both devices.”**

**Line 598: Finally, since we used two-way mixed effects (ICC 3.1 and 3.k) models, our findings are not generalisable (****38****,** **54****). Future studies should replicate and confirm our data across different populations. This includes elite cylists, who are likely to utilise regular strength assessments.**

**However, we acknowledge your feedback and have made some changes, including:**

**Line 103: “Due to poor agreement, the devices are not interchangeable so should be considered device specific. In practice, these preliminary results suggest FF data cannot be compared with B-HHD data and vice versa, while the same device should be used for repeated measurements in this population.”**

**Line 456: “Although both devices initially appear reliable, the observed MDC and agreement values carry potentially important practical and clinical implications.”**

**Line 581: “Due to the observed poor agreement, This means that hip flexion and extension peak force data cannot be interchanged between devices in this population. In practice, this means that measurements should be considered as device-specific, so we cautiously recommend against comparing FF data with B-HHD data (and vice versa), and suggest that the same device should be used for repeated measurements.”**

**Line 604: “This study found that that the MicroFET2 and Forceframe Max have good to excellent reliability and comparable precision for assessing hip flexor and extensor peak force in recreational cyclists. However, their MDC values mean that these devices have potentially limited clinical and practical usefulness in this population. The poor agreement also currently prevents interchangeability between devices. Given the two-way mixed effects ICC models used, further studies are recommended in various populations to confirm these findings.”**

*Some sentences are overly long and could be simplified.*

*Thank you for identifying this. We agree that some sentences could be simplified. As such, we have made the following amendments:*

**Line 453: “This is the first study to evaluate test-retest reliability and agreement between a belt-stabilised, handheld MicroFET2 dynamometer and the Forceframe Max system for measuring peak hip flexion and extension force in cyclists, offering novel data.”**

**Line 539: “This raises some concerns about the sensitivity of both devices to detect subtle but meaningful changes in performance within a session (e.g. following physiotherapy interventions such as manual therapy or proprioceptive taping) or over time as a response to rehabilitation or training.”**

**Line 560: “Despite established concurrent validity between the MicroFET2 and Kanagtech KT360 (****43****), a similar device to the FF, the poor agreement between for hip flexion and extension was surprising, particularly given how far the 95%LOA were exceeded.”**

**Line 526:” However, between-session MDCs were similar (MDC range=41.21-52.61N) and generally consistent with those reported for all externally stabilised portable dynamometers (MDC = 46.7N-49.3N) (****27****), but slightly less than 32% observed for peak torque measurement by McNabb et al (****30****) using a B-HHD. For hip extension, the MDCs observed for both devices (MDCs= 43.71-67.12N ( 20.50-27.80%) were also broadly comparable with these previous reports (****27****,** **30****).”**

*Reviewer #2:*

**Thank you for taking the time to review our manuscript and we appreciate your feedback.**

*Abstract could better highlight the clinical implications of poor agreement (e.g., why interchangeability matters in practice*

**Thank you for highlighting this. As requested, we have added some text to reflect this, which can now be found on line 103:**

**“Due to poor agreement, the devices are not interchangeable so should be considered device specific. In practice, our preliminary results suggest FF data cannot be compared with B-HHD data and vice versa, while the same device should be used for repeated measurements in this populaton.”**

**We have also amended the discussion to reflect this sentence (Line 581):**

**“Due to the observed poor agreement, hip flexion and extension peak force data cannot be interchanged between devices in this population. In practice, this means that measurements should be considered as device-specific; we cautiously recommend against comparing FF data with B-HHD data (and vice versa), and suggest the same device should be used for repeated measurements..”**

*2) Potential bias: hip flexion positions differed (seated vs supine). This introduces systematic variation that may partly explain poor agreement. Needs stronger acknowledgment*

**Thank you for highlighting this. We agree that greater emphasis should be given to the position differences for hip flexion, and we have amended the related text. However, even considering the positional differences, the systematic bias was lower for hip flexion than extension. While we have acknowledged that this is additional source of variation, we have not defined this as systematic. The amended text now states (line 558):**

**“Despite established concurrent validity between the MicroFET2 and Kangatech KT360 (43), a similar device to the FF, the poor agreement between for hip flexion and extension was surprising, particularly given how far the 95% LOA were exceeded. Possible contributing factors include differences in device resolution (FF =1.00N; MicroFET B-HHD= 0.44N) (50, 51) and the potentially greater stability offered by the FF’s rigid aluminium frame and base, compared to the B-HHD’s fabric belt. Specifically for hip flexion, differences in testing positions may have introduced additional variation. Theoretically, the seated position used with the B-HHD may be less stable than the supine position used with the FF. However, this did not appear to systematically bias the measurements, as the mean agreement was low (range = -0.88- 8.22 N). In contrast, there was consistent, larger bias for hip extension, with the FF recording higher peak force (28.04 to 45.38 N). Importantly, across all measurement scenarios presented in Figs 1 and 2, even adjusting values by the largest recorded mean difference (45.38N), the LOA would still exceed the 20N acceptability threshold, making this an unhelpful transformation.”**

*3)Assumption of normality is mentioned but no normality test reported—should be clarified.*

**Thank you for your comment, and we apologise that this is unclear. ICC (3,1) and ICC (3,k) models assume normality of collected data and normally distributed residuals (i.e. the unexplained error terms) (McGraw & Wong (1996)). To address this, we have now included distribution plots for both data types in Appendix S3 and updated the manuscript text accordingly. Importantly, we did not conduct formal significance tests for normality (e.g., Shapiro-Wilk), because the study was** **powered to estimate ICCs precisely, not to test for normality assumptions. Manuscript changes can now be found on line 298:**

**“For within-session analyses, peak force (N) was compared across all three trials for each device, muscle group and limb on each testing day. For all analyses, data distributions were visually inspected using quantile quantile (QQ) plots. For between-session analyses, mean peak force was calculated using all three trials for each participant according to device, muscle group, limb and testing day. Mean values were then compared across testing days.**

**Two-way mixed effects ICC models are required for test-retest designs (****39****) (as used in this study), so within session ICCs_3.1_ and between session ICCs_3.k_ were calculated with corresponding 95% CIs. ICCs were interpreted as poor < 0.40; fair= 0.40- 0.70; good=0.70-0.90; excellent > 0.90 (****40****). Standard error of measurements (SEM) were calculated using the formula** $\boldsymbol{SEM=SD}\sqrt{\boldsymbol{1-ICC}}$**. After ICC model fitting, all residuals were also inspected for normality using histograms. Minimal detectable changes (MDC) were calculated using the formula:** $\boldsymbol{MDC=SEM\times1.96\times}\sqrt{\boldsymbol{2}}$ **(****41****). SEM and MDC values were reported in newtons (N) and as percentages of mean values.”**

**And also on line 334:**

**“For the within-session analyses, descriptive statistics are presented in Table 1. These data and model residuals were approximately normally distributed across devices, limbs and testing days (S3 Appendix). ICCs, 95% CIs, SEMs and MDCs for each limb, device and testing day are presented in Table 2.”**

***References***

Koo, T.K. & Li, M.Y., 2016. A guideline of selecting and reporting intraclass correlation coefficients for reliability research. *Journal of Chiropractic Medicine*, 15(2), pp.155–163. Available at: https://doi.org/10.1016/j.jcm.2016.02.012

Gagnier, J.J., Lai, J., Mokkink, L.B. & Terwee, C.B., 2021. *COSMIN reporting guideline for studies on measurement properties of patient-reported outcome measures*. *Quality of Life Research*, 30(8), pp.2197–2218. Available at: https://doi.org/10.1007/s11136-021-02822-4. [[www.cosmin.nl]](https://www.cosmin.nl/wp-content/uploads/COSMIN-reporting-guideline_1.pdf)
McGraw, K.O. and Wong, S.P., 1996. *Forming inferences about some intraclass correlation coefficients*. Psychological Methods, 1(1), pp.30–46. https://doi.org/10.1037/1082-989X.1.1.30[[2]](https://psycnet.apa.org/record/1996-03170-003)

Mokkink, L.B., Boers, M., van der Vleuten, C.P.M., Patrick, D.L., Alonso, J., Bouter, L.M., de Vet, H.C.W. & Terwee, C.B., 2021. *COSMIN Risk of Bias tool to assess the quality of studies on reliability and measurement error of outcome measurement instruments: User Manual Version 1.0*. Amsterdam UMC, Department of Epidemiology and Data Science. Available at: <https://www.cosmin.nl/wp-content/uploads/user-manual-COSMIN-Risk-of-Bias-tool_v4_JAN_final.pdf>. [[www.cosmin.nl]](https://www.cosmin.nl/wp-content/uploads/user-manual-COSMIN-Risk-of-Bias-tool_v4_JAN_final.pdf)

Mokkink, L.B., Elsman, E.B.M. & Terwee, C.B., 2024. *COSMIN guideline for systematic reviews of patient-reported outcome measures version 2.0*. *Quality of Life Research*, [online] Available at: <https://doi.org/10.1007/s11136-024-03761-6>. [[link.springer.com]](https://link.springer.com/content/pdf/10.1007/s11136-024-03761-6.pdf)

Shrout, P.E. and Fleiss, J.L., 1979. *Intraclass correlations: Uses in assessing rater reliability*. Psychological Bulletin, 86(2), pp.420–428. https://doi.org/10.1037/0033-2909.86.2.420[[1]](https://psycnet.apa.org/record/1979-25169-001)
